# EWAS of Monozygotic Twins Implicate a Role of mTOR Pathway in Pathogenesis of Tic Spectrum Disorder

**DOI:** 10.3390/genes12101510

**Published:** 2021-09-26

**Authors:** Mathis Hildonen, Amanda M. Levy, Christine Søholm Hansen, Jonas Bybjerg-Grauholm, Axel Skytthe, Nanette M. Debes, Qihua Tan, Zeynep Tümer

**Affiliations:** 1 Kennedy Center, Department of Clinical Genetics, Copenhagen University Hospital, Rigshospitalet, 2600 Glostrup, Denmark; mathis.hildonen@regionh.dk (M.H.); marie.amanda.bust.levy@regionh.dk (A.M.L.); 2 Department for Congenital Disorders, Statens Serum Institut, 2300 Copenhagen, Denmark; chsh@ssi.dk (C.S.H.); jogr@ssi.dk (J.B.-G.); 3 Epidemiology and Biostatistics, Department of Public Health, University of Southern Denmark, 5000 Odense, Denmark; askytthe@health.sdu.dk (A.S.); qtan@health.sdu.dk (Q.T.); 4 Tourette Clinics, Department of Paediatrics, Copenhagen University Hospital, 2730 Herlev, Denmark; nanette.marinette.monique.debes@regionh.dk; 5 Unit of Human Genetics, Department of Clinical Research, University of Southern Denmark, 5000 Odense, Denmark; 6 Department of Clinical Medicine, Faculty of Health and Medical Sciences, University of Copenhagen, 2200 Copenhagen, Denmark

**Keywords:** Gilles de la Tourette syndrome, GTS, tics, methylation, epigenetics, TSC1, mTOR, monozygotic twins, chronic tic disorder, tic spectrum disorder

## Abstract

Tic spectrum disorder (TSD) is an umbrella term which includes Gilles de la Tourette syndrome (GTS) and chronic tic disorder (CTD). They are considered highly heritable, yet the genetic components remain largely unknown. In this study we aimed to investigate disease-associated DNA methylation differences to identify genes and pathways which may be implicated in TSD aetiology. For this purpose, we performed an exploratory analysis of the genome-wide DNA methylation patterns in whole blood samples of 16 monozygotic twin pairs, of which eight were discordant and six concordant for TSD, while two pairs were asymptomatic. Although no sites reached genome-wide significance, we identified several sites and regions with a suggestive significance, which were located within or in the vicinity of genes with biological functions associated with neuropsychiatric disorders. The two top genes identified (*TSC1* and *CRYZ*/*TYW3*) and the enriched pathways and components (phosphoinosides and PTEN pathways, and insulin receptor substrate binding) are related to, or have been associated with, the PI3K/AKT/mTOR pathway. Genes in this pathway have previously been associated with GTS, and mTOR signalling has been implicated in a range of neuropsychiatric disorders. It is thus possible that altered mTOR signalling plays a role in the complex pathogenesis of TSD.

## 1. Introduction 

Gilles de la Tourette syndrome (GTS) is characterized by the presence of at least one vocal and two motor tics, while chronic tic disorder (CTD) is characterized by the presence of either vocal or motor tics. Both GTS and CTD are neurodevelopmental disorders with childhood onset (before the age of 18). There is currently no clinical or genetic evidence suggesting that GTS and CTD are separate disorders, and the term tic spectrum disorder (TSD) has been proposed to replace GTS and CTD [1]. For some individuals the symptoms cease as they progress into adulthood. A high proportion of individuals with TSD have comorbid disorders, including attention deficit-hyperactivity disorder (ADHD), obsessive-compulsive disorder (OCD) and autism spectrum disorder (ASD) [1,2,3]. 

TSD is a complex disorder with a largely unknown aetiology, where multiple genes are hypothesized to interact with a range of environmental risk factors. The results of heritability studies suggest genetic risk factors play a substantial role in pathogenesis [4,5], but identification of TSD-associated susceptibility genes has been challenging, likely due to a complex and heterogeneous genetic architecture [6,7,8]. The limited success of the genetic studies can be due to technical issues, such as small sample sizes or incomplete phenotyping of the cohorts, but it is plausible that TSD pathogenesis may be triggered by environmentally mediated epigenetic changes, which may affect gene expression, leading to phenotypic alterations. To date, only two targeted DNA methylation studies of candidate GTS genes has been performed and both studies investigated DNA isolated from peripheral blood lymphocytes. The study carried out by Müller-Vahl and colleagues showed a negative correlation between methylation of the dopamine transporter gene (*SLC6A3*) and tic severity, while higher methylation levels of the dopamine D2 receptor gene (*DRD2*) were associated with GTS and positively correlated with tic severity [9]. The other study carried out by our group did not reveal any differences in methylation levels of the serotonin transporter gene (*SLC6A4*) in GTS individuals compared to controls [10]. 

Studying epigenetic changes is more challenging compared to genomic alterations. Monozygotic (MZ) twin studies can be advantageous to traditional case–control studies, due to the fact that MZ-twins are matched for not only genotype, sex, age and maternal environment, but also partially matched for early environmental influences. As these factors are shown or hypothesized to influence DNA methylation and therefore gene expression, MZ-twin design has a unique advantage [11]. Previously, a single epigenome-wide association study (EWAS) was undertaken in individuals with self-reported tics/tic disorders without reaching a genome-wide significance [12]; however, this study was not designed as a clinical twin-study. In this study, we aimed to investigate disease-associated DNA methylation differences to identify genes and pathways which may be implicated in TSD aetiology. For this purpose, we performed an epigenome wide methylation study in blood lymphocytes of 16 clinically well-defined monozygotic twins, concordant, discordant or asymptomatic for TSD.

## 2. Materials and Methods

### 2.1. Individuals, Phenotyping and Zygosity Studies

Through cross-linking The Danish Psychiatric Central Register and The Danish Twin Register we identified 204 twin pairs, where at least one twin had GTS or CTD (Collectively referred to as TSD throughout the manuscript). All the twins or legal guardians were contacted, and 56 twin pairs accepted to participate. DNA was isolated from peripheral blood and the zygosity of the twins was determined with SNP genotyping using Infinium Global Screening Array (llumina). Fourteen twin pairs were monozygotic. Interviews were carried out with all the twins and a trained neuropediatrician made the diagnosis using the DSM-V (Diagnostic and Statistical Manual of Mental Disorders-V) criteria. Eight of these pairs were discordant for TSD and six were concordant. Furthermore, two asymptomatic MZ twin pairs were included in the study. The study was approved by the Danish Institutional Review Board (2011 H-2-2010-144).

### 2.2. DNA Methylation Profiling and Quality Control

Genomic DNA was extracted from peripheral blood and bisulfite treatment was carried out using standard protocols. Infinium MethylationEPIC arrays (Illumina, San Diego, CA, USA) were used to analyse the bisulfite converted DNA according to manufacturer’s protocol. A detection *p*-value for quality control (QC) was calculated using the free R package minfi [13]. Probes with detection *p* > 0.01, probes on sex chromosomes, probes harbouring single nucleotide polymorphisms (SNPs) and probes with known cross-reactivity were excluded from the analysis. After QC, 756,887 probes remained for analysis. Quantile normalization implemented in the R package minfi was used for data normalisation. β values defined by Illumina’s formula as β = M/(M + U) was used to quantify DNA methylation levels (M, methylated and U, unmethylated signal intensities at each CpG site). To conduct statistical analysis methylation β values were logit transformed to M values.

### 2.3. Statistical Analyses

The DNA used in this study was extracted from whole blood, which contains several different types of blood cells. As different cell types are known to have different methylation patterns, the R package FlowSorted.Blood.EPIC [14] was used to estimate the proportion of CD4T, CD8T, NK cells, B cells, monocytes and neutrophils in each sample. The Mann-Whitney U-test was used to compare the cell type composition between TSD individuals and controls. 

A linear mixed model using the R package lmerTest [15], regressing methylation levels on clinical phenotype was employed to identify differentially methylated positions (DMPs) associated with TSD. The model was corrected for age, with twin pairing as a random effect. As we did not observe any difference in cell-type composition between TSD individuals and controls (Supplementary Figure S1), and as our sample size was relatively small, we did not include cell-type composition in the analysis to avoid overfitting the model. False discovery rate (FDR) was set to <0.05 as a cut-off for genome-wide significance to correct for multiple testing. DMPs significantly associated with TSD (*p* < 0.01) were submitted to the Genomic Regions Enrichment of Annotations Tool (GREAT 3.0), an online tool which annotates genomic positions to genes and cis-regulatory elements, and identifies enriched gene ontologies and pathways.

In addition to investigating methylation differences at single sites, we also employed a region-based approach using the python library comb-p [16]. This identifies differentially methylated regions (DMRs) by calculating autocorrelation, combining *p*-values of adjacent sites and assigning significance to the regions identified. We used the results from the linear mixed model, with an unadjusted *p* of <1 × 10^−^^3^ to seed a region, and a maximum distance of 500 bp between the CpG sites. The region results were corrected for multiple testing using the Šidák correction.

## 3. Results 

We did not observe any difference in cell-type composition between TSD individuals and controls (Supplementary Figure S1).

**Figure 1:**
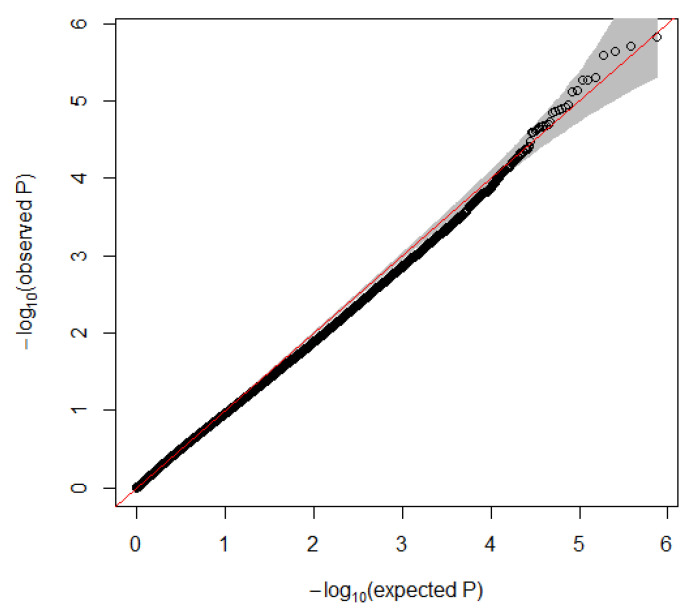
Quantile-quantile plot of expected (*x*-axis) versus observed (*y*-axis) *p*-values. At lower *p*-values there is a slight deviation from the expected pattern which may indicate presence of non-random association. The circles represent the CpG sites investigated in this study. The red line indicates the expected pattern if there is no difference between expected and observed *p*-values, and the grey area indicates a confidence interval of 95%. log_10_, logarithm base 10.

**Figure 2:**
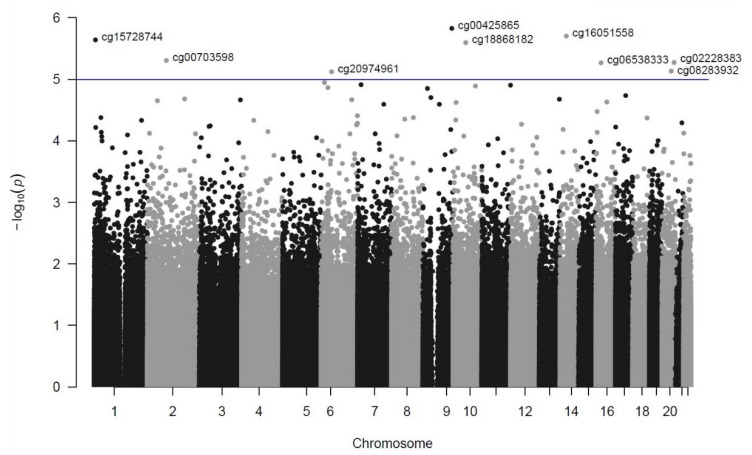
The Manhattan plot shows the *p*-value and chromosomal position of all CpG sites investigated. Each dot represents a CpG site, and the horizontal blue line denotes the threshold (*p* = 1 × 10^−^^5^) for suggestive significance. Sites with suggestive significance are annotated with Illumina CpG loci ID. Negative logarithm base 10 (−log_10_) of the *p* value is shown on the *y*-axis and chromosome positions on the *x*-axis.

To identify differentially methylated probes in TSD individuals we employed a linear mixed model corrected for age with twin pairing as a random effect. None of the probes reached genome-wide significance defined as FDR < 0.05. As illustrated in Figure 1, a slight deviation from the expected *p*-values was observed, which could indicate non-random association. Nine sites with suggestive significance (*p* < 1 × 10^−^^5^) were detected (Table 1, Figure 2). Three of these sites were located within or close to genes (*TSC1*, *NEMF* and *ADA*) which were previously implicated in with neuropsychiatric disorders. The DMP most significantly associated with TSD was located in the promoter region of *TSC1*.

DMPs associated with TSD (*p* < 0.01, *n* = 5707) were investigated for enrichment of gene ontologies and pathways using GREAT. Genomic positions of 5676 DMPs were annotated to one or more genes and/or cis-regulatory elements, and four gene ontologies and two pathways were found to be enriched (Table 2). The genes identified in the enrichment analysis are listed in Supplementary Table S1.

We combined *p*-values of adjacent sites identified by the linear mixed model in the site-specific analysis to identify differentially methylated genomic regions in TSD individuals compared to controls. Seven regions had three or more CpG sites and a Šidák corrected *p*-value below 0.5. Two regions had a corrected *p*-value below 0.1 (Table 3).

## 4. Discussion 

In this study we have investigated genome-wide methylation patterns in 16 monozygotic twin pairs, of which eight were discordant and six concordant for TSD, while two pairs were asymptomatic. None of the investigated CpG sites reached a genome wide significance, but we identified nine CpG sites with suggestive significance (*p* < 1 × 10^−^^5^, Table 1) and seven DMRs (Šidák-corrected *p* < 0.5, Table 3), which may be associated with TSD. 

Of the nine DMPs with suggestive significance three were annotated to genes previously associated with neuropsychiatric disorders (*TSC1*, *NEMF* and *ADA*). *NEMF* (nuclear export mediator factor) encodes a component of the ribosome quality control complex and has previously been associated with ASD [17]. Furthermore, pathogenic variants of *NEMF* have been shown to cause neurodegeneration in mice [18]. *ADA* (adenosine deaminase) encodes a protein which is important for purine metabolism, and *ADA* deficiency causes severe combined immunodeficiency (SCID). Reduced adenosine deaminase activity has been observed in ASD individuals [19] and a variant of the gene with low enzymatic activity has been associated with ASD [20]. 

Of the seven DMRs three were annotated to the regulatory regions of genes previously associated with neuropsychiatric disorders (*SDK1*, *CHD2* and *CLN8*). *SDK1* (The Sidekick Cell Adhesion Molecule 1) has been associated with ASD [21,22,23,24], and ADHD [25,26]; *CHD2* (Chromodomain Helicase DNA Binding Protein 2) variants has been identified in individuals with central nervous system pathologies [27]; and *CLN8* (the ceroid-lipofuscinosis, neuronal 8) has been linked to ASD through rare missense variants found in a Japanese family [28].

The DMP most significantly associated with TSD was within the promoter region of *TSC1* coding for a subunit of the tuberous sclerosis complex (TSC). Pathogenic *TSC1* variants lead to tuberous sclerosis, which is characterized by skin abnormalities, developmental delay, epilepsy and behavioural problems such as ADHD and ASD [29]. TSC1 and TSC2 together with TBC1D7 (TBC1 domain family, member 7) comprise the tuberous sclerosis protein complex which has crucial roles in cell growth. Notably, in a GWAS (genome-wide association study) meta-study investigating the shared common background of GTS and ADHD the top and third SNPs were annotated to *TBC1D7* [30]. TSC1 regulates signalling of mTOR (mechanistic target of rapamycin) by the dual function of inhibiting the mTOR complex 1 and activating the mTOR complex 2 [31,32,33]. mTOR functions as a serine/threonine protein kinase which promotes synthesis of lipids, nucleotides and proteins, and it also comprises part of the larger PI3K/AKT/mTOR pathway, which is associated with neuropsychiatric disorders such as depression and ASD [34,35,36]. Disruption of mTOR signalling can affect neuronal growth and proliferation, as well as the release of dopamine [33,37], which has extensively been studied in GTS [38,39]. Altered mTOR signalling has also been associated with ASD, schizophrenia, depression and epilepsy [40,41,42,43,44]. An increased hazard ratio (HR) for epilepsy has been observed in children with GTS (adjusted HR = 16.27, 95% CI = 6.26–18.46), which may indicate a neurobiological overlap between TSD and epilepsy [45]. However, in another study a negative, though not significant, genetic correlation was found between GTS and epilepsy [46].

Genes involved in the phosphoinositide and the PTEN pathways, as well as insulin receptor substrate binding, was significantly enriched among the nominally significant DMPs (*p* < 0.01) we identified in this study (Table 2). These pathways and cellular processes also constitute parts of the larger PI3K/AKT/mTOR pathway. SNPs located in the receptor tyrosine kinase gene *FLT3*, which is an activator of the PI3K/AKT/mTOR pathway [47], have been found in a GTS-pathway investigation and a GTS-GWAS [48,49]; and it was the only SNP reaching genome-wide significance in the latter study [49]. Furthermore, through exome sequencing of sporadic GTS individuals and their healthy parents, a likely pathogenic missense variant (rs140964083: G>A) was identified in *RICTOR* (rapamycin-insensitive companion of mammalian target of rapamycin) encoding a component of the mTOR2 complex [50].

The top DMR in the present study was within the overlapping first exons of *CRYZ* (Crystallin Zeta) and *TYW3* (TRNA-YW Synthesizing Protein 3 Homolog) which are transcribed from opposite strands. In a previous GWAS, a SNP near these two genes was significantly associated with resistin levels in blood and transcript levels of the gene encoding resistin in leukocytes [51]. Notably, increased resistin levels have been shown to activate mTOR signalling through phosphorylation of TSC2 [52]. 

In conclusion, the results of the present study and the findings from previous studies suggest that deregulation of the PI3K/AKT/mTOR pathway may be one of the contributing risk factors in TSD pathogenesis. Involvement of this pathway and particularly mTOR signalling are widely recognized as contributing factors to a broad range of neuropsychiatric disorders, which further support this hypothesis. As TSD is considered a multifactorial disease, the pathogenesis is likely to include a combination of genetic, epigenetic and environmental factors, and to consist of several pathways and cellular functions. However, further methylation studies using larger cohorts should be carried out to replicate the current findings, and expression studies of the components of the PI3K/AKT/mTOR pathway may give insights to the TSD pathogenesis.

The present study is the first to date epigenome-wide study of methylation changes in monozygotic twins with TSD. The relatively small sample size was a limitation, but the twin-based design offers unique advantages over the classic case-control setup even with a relatively small sample size. We carried out the methylation studies using peripheral blood, and it is possible that brain regions known to be involved in TSD pathology may show different methylation patterns to the ones we observed. Epigenomic studies of neuropsychiatric disorders have largely been carried out on peripheral blood, as brain tissue is not available. However, studies suggest that blood methylation can be informative for psychiatric conditions, as factors affecting the brain have been shown to leave biomarker signatures in the blood. Furthermore, one of the significant findings of the GTS-pathway study implicated involvement of lymphocytic pathways in GTS aetiology, which supports that blood can be used to identify methylation biomarkers. 

## Figures and Tables

**Table 1:** Differentially methylated positions (DMPs) with a *p*-value < 1 × 10^−^^5^.

Rank	DMP (CpG Site)	Chromosome Position (GRCh37/hg19)	*p*-Value	FDR	Coefficient	Closest Genes (Distance from TSS in bp) ^a^
1	cg00425865	chr9:135820111	1.49 × 10^−^^6^	0.48	−0.6889	*TSC1* (−104)/*GFI1B* (−826)
2	cg16051558	chr14:50235537	1.98 × 10^−^^6^	0.48	0.3805	*KLHDC2* (+1212)/*NEMF* (+84,383)
3	cg15728744	chr1:5917533	2.29 × 10^−^^6^	0.48	0.5383	*NPHP4* (+134,997)
4	cg18868182	chr10:60086874	2.54 × 10^−^^6^	0.48	0.1460	*UBE2D1* (−7860)/*CISD1* (+58,057)
5	cg00703598	chr2:88480233	4.94 × 10^−^^6^	0.58	−0.2805	*THNSL2* (+9255)/*TEX37* (−343,935)
6	cg02228383	chr20:56934684	5.33 × 10^−^^6^	0.58	0.5032	*VAPB* (−29,493)/*RAB22A* (+49,933)
7	cg06538333	chr16:23445078	5.39 × 10^−^^6^	0.58	−0.6260	*COG7* (+19,422)/*SCNN1B* (+131,488)
8	cg08283932	chr20:43280707	7.36 × 10^−^^6^	0.63	−0.6783	*ADA* (−325)
9	cg20974961	chr6:49500569	7.54 × 10^−^^6^	0.63	−0.3322	*GLYATL3* (+32,899)/*RHAG* (+103,982)

The table shows the identified DMPs with a significance level <1 × 10^−^^5^, their chromosome position, significance values, coefficient and closest genes. a, ‘+’ indicates that the DMP is downstream of the TSS, ‘−’ indicates that the DMP is upstream of the TSS; FDR, false discovery rate; TSS, transcription start site.

**Table 2:** Enrichment of gene ontologies and pathways among DMPs with *p* < 0.01.

Ontology	Term	Binom Raw *p*-Value	Binom FDR *q*-Value	Binom Fold Enrichment	Binom Observed Region Hits
GO Cellular Component	Insulin receptor substrate binding	3.88 × 10^−4^	4.33 × 10^−2^	2.25	23
GO Biological Process	Positive regulation of hormone metabolic process	9.20 × 10^−6^	1.12 × 10^−3^	2.70	26
	Positive regulation of hormone biosynthetic process	1.56 × 10^−5^	1.79 × 10^−3^	3.08	20
	Regulation of protein ubiquitination involved in ubiquitin-dependent protein catabolic process	5.10 × 10^−4^	2.65 × 10^−2^	2.36	20
MSigDB Pathway	Phosphoinositides and their downstream targets	7.62 × 10^−4^	3.14 × 10^−2^	2.07	25
	PTEN is a tumor suppressor that dephosphorylates the lipid messenger phosphatidylinositol triphosphate	1.13 × 10^−3^	3.73 × 10^−2^	2.39	17

The table shows the results of the enrichment analysis of the nominally significant DMPs (*p* < 0.01). Significance levels are reported as binomial raw *p*-values and FDR-adjusted *q*-values. GO, gene ontology; MSigDB, Molecular Signatures Database.

**Table 3:** Differentially methylated regions (DMRs) in TSD individuals.

Rank	Chromosome Position (GRCh37/hg19)	Length (bp)	No. of Sites	SLK adj. *p*-Value	Šidák adj. *p*-Value	Closest Genes (Distance to TSS) ^a^
1	chr1:75198768-75199178	410	8	7.60 × 10^−6^	0.001	*CRYZ* (+119)/*TYW3* (+133)
2	chr7:3227262-3227333	71	3	9.01 × 10^−6^	0.091	*SDK1* (−113,782)/*CARD11* (−143,719)
3	chr3:138067848-138068014	166	6	3.07 × 10^−5^	0.131	*MRAS* (+1392)/*ESYT3* (−85,524)
4	chr15:93353059-93353199	140	3	7.35 × 10^−5^	0.328	*CHD2* (−89,929)/*FAM174B* (−153,941)
5	chr19:17830341-17830453	112	3	7.61 × 10^−5^	0.402	*MAP1S* (+236)
6	chr13:41635362-41635513	151	4	1.05 × 10^−4^	0.409	*WBP4* (+28)
7	chr8:1765217-1765388	171	7	1.54 × 10^−4^	0.494	*ARHGEF10* (−6839)/*CLN8* (+53,375)

The table shows the DMRs with a Šidák adjusted *p*-value < 0.5, their length (in bp and number of CpG sites), significance and closest genes. a, ‘+’ indicates that the DMR is downstream of the TSS, ‘−’ indicates that the DMR is upstream of the TSS; SLK, Stouffer-Liptak-Kechris; adj, adjusted; TSS, transcription start site.

## Data Availability

The data presented in this study are available on request from the corresponding author.

## Supplementary Materials

The following are available online at https://www.mdpi.com/article/10.3390/genes12101510/s1, Figure S1: Blood cell composition of samples, Table S1: Genes identified in the enrichment analysis.
